# Non-Coding RNA and Tumor Development in Neurofibromatosis Type 1: *ANRIL* Rs2151280 Is Associated with Optic Glioma Development and a Mild Phenotype in Neurofibromatosis Type 1 Patients

**DOI:** 10.3390/genes10110892

**Published:** 2019-11-05

**Authors:** Viviana Tritto, Luca Ferrari, Silvia Esposito, Paola Zuccotti, Donatella Bianchessi, Federica Natacci, Veronica Saletti, Marica Eoli, Paola Riva

**Affiliations:** 1 Department of Medical Biotechnology and Translational Medicine, Università degli Studi di Milano, via F.lli Cervi 93, Segrate, 20090 Milan, Italy; viviana.tritto@unimi.it (V.T.); luca.ferrari@unimi.it (L.F.); paola.zuccotti@gmail.com (P.Z.); 2 Unit of Developmental Neurology, Fondazione I.R.C.C.S. Istituto Neurologico C. Besta, via Celoria 11, 20133 Milan, Italy; Silvia.Esposito@istituto-besta.it (S.E.); Veronica.Saletti@istituto-besta.it (V.S.); 3 Unit of Molecular Neuro-Oncology, Fondazione I.R.C.C.S. Istituto Neurologico C. Besta, via Celoria 11, 20133 Milan, Italy; Donata.Bianchessi@istituto-besta.it; 4 Unit of Medical Genetics, Fondazione I.R.C.C.S. Ca’ Granda Ospedale Maggiore Policlinico, via della Commenda 12, 20122 Milan, Italy; federica.natacci@policlinico.mi.it

**Keywords:** neurofibromatosis type 1, ncRNA, tumor development, optic glioma, mild NF1 phenotype, *ANRIL* variants

## Abstract

Non-coding RNAs (ncRNAs) are known to regulate gene expression at the transcriptional and post-transcriptional levels, chromatin remodeling, and signal transduction. The identification of different species of ncRNAs, microRNAs (miRNAs), circular RNAs (circRNAs), and long ncRNAs (lncRNAs)—and in some cases, their combined regulatory function on specific target genes—may help to elucidate their role in biological processes. NcRNAs’ deregulation has an impact on the impairment of physiological programs, driving cells in cancer development. We here carried out a review of literature concerning the implication of ncRNAs on tumor development in neurofibromatosis type 1 (NF1), an inherited tumor predisposition syndrome. A number of miRNAs and a lncRNA has been implicated in NF1-associated tumors, such as malignant peripheral nerve sheath tumors (MPNSTs) and astrocytoma, as well as in the pathognomonic neurofibromas. Some authors reported that the lncRNA *ANRIL* was deregulated in the blood of NF1 patients with plexiform neurofibromas (PNFs), even if its role should be further elucidated. We here provided original data concerning the association of a specific genotype about *ANRIL* rs2151280 with the presence of optic gliomas and a mild expression of the NF1 phenotype. We also detected the LOH of *ANRIL* in different tumors from NF1 patients, supporting the involvement of *ANRIL* in some NF1-associated tumors. Our results suggest that *ANRIL* rs2151280 may be a potential diagnostic and prognostic marker, addressing early diagnosis of optic glioma and predicting the phenotype severity in NF1 patients.

## 1. Introduction

In recent years, an increasing number of functions for small non-coding RNAs (sncRNAs) has been identified. They are key molecules mediating fundamental cellular processes, such as transcription, post-transcriptional modifications, chromatin remodeling, and signal transduction. Dysfunction in an increasing number of microRNAs (miRNAs) has been reported during carcinogenesis, indicating a main role of these molecules in the deregulation of target genes involved in the control of cell growth and proliferation [1].

Next-generation sequencing (NGS) technologies allow the identification of different species of non-coding RNAs (ncRNAs), some highly conserved, such as miRNAs [2] and circular RNAs (circRNAs), and others like long ncRNAs (lncRNAs) that generally show a lower level of conservation [3]. 

NcRNAs constitute most of the transcriptional output in human cells, and they have been shown to play a fundamental role in cellular processes, both in physiological and pathological contexts [4,5]. 

Studies of more than two decades of miRNAs led to considering the function of these molecules within a network rather than singularly. In fact, an miRNA targets the mRNAs of many genes, and an mRNA of each gene can be targeted by multiple miRNAs, making these miRNAs and mRNAs associated into a common regulatory network [6,7]. We know that this complexity is even higher if we consider the contribution of additional regulatory elements that regulate mRNA stability—in particular, those located in the 3′ untranslated region (UTR) of many transcripts [8,9].

Moreover, miRNAs may interact with circRNAs and lncRNAs, controlling their stability, and lncRNAs and circRNAs regulate the level of miRNAs available in a cell by mechanisms of sequestering or releasing of the specific miRNA molecules. The complexity of these interactions suggests that they play a key role in the regulation of important cellular and molecular pathways.

It is expected that whenever these interactions are altered, widespread consequences affect cell fate by transforming the transcriptome and leading to a pathological condition common to cancer [1].

This review is focused on the identification of the role of ncRNAs in relation to tumor susceptibility in patients affected by neurofibromatosis type 1 (NF1). 

NF1 is a familial tumor predisposition syndrome characterised by the onset of both benign and malignant tumors located in the peripheral and central nervous system. This disease is caused by *NF1* (neurofibromin 1) inactivating germline mutations [10], leading to the development of multiple benign cutaneous neurofibromas in most patients. A subgroup ranging from 30% to 50% is affected by large plexiform neurofibromas, and about 10% of them develop malignant peripheral nerve sheath tumors (MPNSTs). These tumors, characterised by high-grade cancer cells, bring out a critical diagnostic as well as therapeutic challenge for these patients, and about 50% of MPNSTs are diagnosed in association with NF1 [11]. Due to the lack of an effective treatment, these NF1 patients display a poor prognosis.

The mechanisms of MPNST tumorigenesis have been poorly described. Besides the biallelic *NF1* gene inactivation necessary for tumor development, it is currently known that further molecular alterations, such as epigenetic changes (often involving miRNAs), may promote uncontrolled tumor growth. Little is known about the role of ncRNAs—in particular, miRNAs and lncRNAs mainly involved in cancer—on benign and malignant tumor development in relation to NF1.

Interestingly, the polymorphism rs2151280 located in the lncRNA *ANRIL* (*CDKN2B antisense RNA 1*) [12] was associated with the number of plexiform neurofibromas (PNFs) in NF1 patients, and 9p21.3 deletions, including the *CDKN2A/B–ANRIL* locus, are detected in PNFs [13]. *ANRIL* is often deregulated in cancer, and some variants have been associated with some tumors, including gliomas [14].

We here report a review of literature in this field, besides providing our original data concerning the association of a specific genotype of *ANRIL* rs2151280 with the presence of optic gliomas and a mild expression of the disease, according to Riccardi and Ablon scores. *ANRIL*, mapped at the 9p21.3 region, expresses an lncRNA, and is often deregulated in cancer [12]. We also detected the 9p21.3 loss of heterozygosity (LOH) in different tumor types of NF1 patients, supporting the involvement of *ANRIL* in some NF1-associated tumors.

## 2. Materials and Methods

### 2.1. Review of Literature

The review of literature was carried out by considering the main relevant original works and reviews in the field concerning ncRNAs involved in tumor development in neurofibromatosis type 1, and distinguishing their implications for pathognomonic neurofibromas and specific tumors associated with the disease (MPNST, pilocytic astrocytoma, optic glioma).

### 2.2. Human Subjects

The investigations were carried out following the rules of the Declaration of Helsinki of 1975, revised in 2013. This study is part of a research grant approved by the Italian Health Minister (RFINN-2008-1204836), and according to the Italian regulations, it did not require specific ethical approval before undertaking the research, because it only uses anonymous data collected during routine patient care. The results were submitted and approved by ethical committee of Regione Lombardia, a section of C. Besta Neurological Institute Foundation.

Written informed consent was given by both the patients and subjects recruited as unaffected controls. Additional informed consent was obtained from all of the subjects who may be identified on the bases of the information contained in this article.

### 2.3. Patients and Phenotypic Data

One hundred and four unrelated, consecutive patients with NF1, on whom we performed molecular *NF1* genetic testing at our institute between January 2013 and September 2014, were included in the study. In all, the diagnoses fulfilled National Institutes of Health (NIH) diagnostic criteria, and in 100 of them a pathogenic *NF1* mutation was identified. The four patients in which *NF1* mutation was not found were all adults with a certain NF1 diagnosis. The lack of *NF1* mutation identification is expected in about 5% of patients; in these cases, the mutation is hypothesized to localize in one of the *NF1* introns [15]. The median age was 35 (2–71). Eligible patients were identified using an electronic patient database search request. All medical records were surveyed, and the following data were collected at the time of mutation analysis and re-verified for accuracy at the time of this study: data of birth; gender; age at the time of genetic testing; mode of inheritance; and NF1 signs and symptoms, including visual impairment, pain, epilepsy, cognitive impairment, plexiform and spinal neurofibromas, optic nerve glioma, and other neoplasms of the central nervous system and other organs. Two different medical severity scales [16,17] were used to assess overall disease severity and to rate the visibility features of the disease (Tables S1 and S2). All patients underwent brain and spinal MRI (magnetic resonance imaging) scans. NF1 optic pathway glioma (OPG) cases were defined as patients diagnosed with NF1 and an OPG, confirmed by MRI scan. Spinal neurofibroma NF1 cases were defined as patients with at least one neurofibroma along the spinal root.

### 2.4. DNA and RNA Extraction

Constitutional DNA and total RNA extracted from peripheral blood samples were conserved in ethylenediaminetetraacetic acid (EDTA). Constitutional DNA was extracted using a NucleoSpin Blood Kit (Macherey-Nagel, Duren, Germany) in accordance with the manufacturer’s instructions and stored at a temperature of −20 °C; RNA was extracted using an RNeasy Mini Kit (Qiagen, Hilden, Germany) and stored at a temperature of −80 °C. DNA from tumor samples was extracted by means of Trizol reagents (Thermo Fisher, Waltham, MA, United States) following manufacturer’s instructions.

### 2.5. Genotyping of Single Nucleotide Polymorphism Rs2151280

The genotype of the single nucleotide polymorphism (SNP) rs2151280, located within intron 3 of *ANRIL*, was investigated by PCR amplification of the genomic region and subsequent sequence analysis of PCR fragments, amplified from blood-derived DNA of the 104 NF1 patients. PCR was performed by GoTaq DNA Polymerase (Promega, Fitchburg, WI, United States) following manufacturer’s instructions, with the primer shown in Table S3.

### 2.6. Sequencing Analysis

The PCR products were bi-directionally sequenced using the Terminator v3.1 Cycle Sequencing Kit (Thermo Fisher, Waltham, MA, United States), and resolved on an automated ABI-3130xl DNA genetic analyser (Thermo Fisher, Waltham, MA, United States). The output data were analysed using SeqScape software v2.5 (Thermo Fisher, Waltham, MA, United States).

### 2.7. Reverse Transcription (RT) and Quantitative Real-Time PCR (qPCR)

One microgram of total RNA was reverse transcribed using the iScriptTM cDNA Synthesis Kit (Bio-Rad Laboratories Inc., Berkeley, CA, United States). The specific oligonucleotides used to amplify the *CDKN2A* (*cyclin dependent kinase inhibitor 2a*), *CDKN2B* (*cyclin dependent kinase inhibitor 2b*), *ARF*, and *ANRIL* transcripts are shown in Table S3; *TBP* (*TATA-box binding protein*) was used as a housekeeping control. The qPCR assays were made using GoTaq-qPCR master mix (Promega) and run on an iQ5 Real-Time ThermalCycler (Bio-Rad Laboratories Inc., Berkeley, CA, United States). 

### 2.8. LOH Analysis

Microsatellite analysis was performed on genomic DNA extracted from peripheral blood mononuclear cells (PBMCs) and from tumor samples, amplified by means of PCR using a carboxyfluorescein- or hexachlorofluorescein-labelled forward primer for each marker (Sigma Aldrich, St. Louis, MO, United States) and GoTaq DNA Polymerase (Promega, Fitchburg, WI, United States) following reported procedures [18]. The DNA fragments were separated by means of capillary electrophoresis (ABI 3130XL, Thermo Fisher, Waltham, MA, United States) and analyzed using Genemapper software (version 3.1, Thermo Fisher, Waltham, MA, United States). Oligonucleotide sequences are reported in Table S3.

### 2.9. Statistical Analysis

Descriptive and frequency statistical analyses were carried out, and comparisons were made using the SPSS 17.0 software. Chi-square or Fisher exact tests were used to examine the differences in categorical variables between groups. Genotype–phenotype correlations were studied using a multiple logistic regression model odds ratio (OR), and 95% confidence intervals were calculated when there was a significant effect. A *p* value (*p*) < 0.05 was considered as statistically significant. The resulting *p* values were adjusted for multiple comparisons using the Benjamin–Hochberg (B_H) procedure, with a false discovery rate of 0.2.

## 3. Results

### 3.1. Non-Coding RNA and Tumor Susceptibility in Neurofibromatosis Type 1

Some studies report on lncRNA and miRNA network involvement in different molecular mechanisms of gene expression modulation, associated with tumor development and progression in NF1 patients [1].

The increased risk of MPNST development by NF1 patients is well documented. MPNST is a rare and often aggressive soft tissue sarcoma that originates from the peripheral nerve. In 80% of cases, MPNSTs arise from pre-existing plexiform neurofibromas (PNFs) that undergo several steps of malignant transformation in Schwann cells. The first step is the complete *NF1* loss of function via biallelic-inactivating mutations, as well as subsequent hyperactivation of RAS and its downstream effector pathways. The next steps in the malignant progression are the deletion of cell-cycle regulator *CDKN2A/B* genes and loss-of-function mutations in the tumor suppressor *TP53* (*tumor protein p53*), as well as in the histone methyltransferase polycomb repressive complex 2 (PRC2), including its core components EED (Embryonic Ectoderm Development) or SUZ12 (SUZ12 Polycomb Repressive Complex 2 Subunit) [19,20]. The emerging role of miRNAs in tumorigenesis led the researchers to study their implication in tumor development in NF1 patients. Masliah-Planchon et al. applied an RT-PCR analysis to comprehensively quantify the expression of 377 miRNAs in a large panel of dermal neurofibromas (DNFs), plexiform neurofibromas, and MPNSTs, as well as in two adult peripheral nerves as a non-tumorigenic control tissue and in two MPNST cell lines as malignant controls. Investigating differentially expressed miRNAs between PNFs and MPNSTs, they found in MPNSTs 103 significantly upregulated and ten downregulated miRNAs [21]. Previous studies had already associated these miRNAs to RAS-MAPK (Mitogen-Activated Protein Kinase) pathway regulation (Let-7b, miR-10b, and miR-195) [22,23,24], *PTEN* (*phosphatase and tensin homolog*) inhibition (miR-19a, miR-106b, and miR-301a) [25,26,27], epithelial–mesenchymal transition (let-7b, miR-9, miR-135a, miR-135b, and miR-200c) [21,28,29,30,31], *HOX* gene expression (miR-9, miR-10a, miR-10b, miR-196b, and miR-210) [32,33,34,35], and cell cycle regulation (let-7b, miR-20a, miR-106b, miR-129-3p, miR-195, and miR-210) [35,36,37,38,39]. This study aimed to identify an miRNA signature that could represent a useful complement to the NF1-associated tumor diagnosis and prognosis, and a novel strategy for effective pharmacological therapies of NF1 tumors [21]. 

Two miRNAs have been directly implicated in MPNST development by miRNA expression profiling studies on peripheral nerve sheath tumor samples: miR-34a and miR-214. miR-34a is a tumor suppressor downregulated in MPNSTs, as shown by in vitro studies using the cell lines MPNST-14 (NF1 mutant) and MPNST-724 (from a non-NF1 individual). The results showed that exogenous expression of p53 or miR-34a promotes apoptotic cell death, and exogenous expression of p53 in MPNST cells induces miR-34a and other miRNAs; therefore, p53 inactivation and the following loss of expression of miR-34a may significantly contribute to MPNST development. As a result, the authors propose this miRNA as a candidate therapeutic treatment target in MPNSTs [40]. As it is the most overexpressed miRNA in MPNSTs, miR-34a is an oncogene. Other studies performed on mouse neural cells have shown that *TWIST1* (*twist family BHLH transcription factor 1*), a regulator of metastasis highly expressed in the majority of MPNSTs, induces miR-214 expression [41].

Gong et al., performing an analysis of miRNA expression by microarray and qRT-PCR, found in NF1 and non-NF1 MPNST tumor tissues, and in tumor cell lines, the downregulation of miR-204, which is located at a cancer-associated genomic region showing a high frequency of LOH in tumors. The authors demonstrated that by restoring miR-204 expression, cellular proliferation, migration, and invasion were decreased in NF1 and non-NF1 MPNST cell lines in vitro, and tumor growth and malignant progression were reduced in non-NF1MPNST cell lines in vivo. These findings support the hypothesis that miR-204 is a tumor suppressor involved in MPNST tumorigenesis and progression, and may represent a novel biomarker for diagnosis and a possible therapeutic treatment target in MPNSTs [42].

Another miRNA that plays an important role in MPNST development is miR-21, identified as an oncogene overexpressed in MPNSTs having *PDCD4* (*programmed cell death protein 4*) as the target gene. Itani et al. demonstrated that miR-21 expression level was significantly higher in MPNSTs than that in neurofibromas (*p* < 0.05), applying miRNA expression profile analysis and quantitative real-time reverse transcription PCR on MPNSTs, neurofibromas, and normal nerves in clinical samples and on three MPNST cell lines. In addition, the authors demonstrated the important role of miR-21 in MPNST progression via transfection of an miR-21 inhibitor in one MPNST cell line. These cells underwent apoptosis, indicating that miR-21 and its target PDCD4 may be candidate therapeutic targets against MPNST progression [43].

Presneau et al. identified 16 significantly differentially expressed miRNAs in MPNSTs compared with neurofibroma, using a microarray analysis on NF1 patients’ samples and reverse transcription quantitative PCR to validate the results. Of these, 14 were downregulated (miR-30e*, miR-29c*, miR-29c, miR-340*, miR-30c, miR-139-5p, miR-195, miR-151-5p, miR- 342-5p, miR-146a, miR-150, miR-223, let-7a, and let-7g), and two were upregulated (miR-210 and miR-339-5p) in MPNSTs. Then the authors focused on miR-29c, because its target genes are all involved in cell migration and invasion. Functional studies in an MPNST cell line showed that downregulation of miR-29c, using a mimic of the mature miR-29c, increased the invasive and migratory capacities of nerve sheath tumor cells; thus, a novel therapeutic approach against MPNSTs could be to restore miR-29c expression in these tumors [44].

NF1 patients also have a high risk of developing gliomas [45], which are among the most common types of brain cancer and arise from the supportive (“gluey”) tissue of the brain—precisely, from glial cells. A glioma is another tumor in which altered miRNA expression plays an important role, through the regulation of tumorigenic processes, such as receptor tyrosine kinase signaling, suppression of differentiation, cell cycle stimulation, apoptosis inhibition, invasion, and angiogenesis. The most frequent form of glioma in the pediatric age is the pilocytic astrocytoma (PA), a low-grade neoplasm that occurs frequently in children and young adults, whose tumorigenesis has been associated with *BRAF* (*B-Raf proto-oncogene, serine/threonine kinase*) mutations and fusion, but also with miRNA action. Ho et al., investigating different PA groups by the analysis of differentially expressed miRNAs in NF1-associated tumors versus tumors with *BRAF* alterations, found four miRNAs differentially expressed between NF1-associated and *BRAF* fusion-positive tumors: in NF1 tumors, hsa-miR-650 and hsa-miR-1276 were overexpressed, whereas hsa-miR-744* and hsa-miR-187* were under-expressed [46]. In addition, Darrigo et al. revealed a subset of 30 under-expressed miRNAs in PA samples of patients with NF1, evaluating global miRNA expression in 30 micro-dissected samples, including pediatric PAs, NF1-associated PAs, and cerebral white matter by the microarray method. These different miRNA profiles of NF1-associated PAs were previously related with known deregulated pathways in cancer, such as the cell cycle and hippo pathways, and suggest a distinct tumorigenesis process associated with miRNA dynamics in this PA subgroup [47]. Table S4 summarizes the reported expression level of miRNAs analyzed in tumors developed by NF1 patients.

### 3.2. Non-Coding RNA and Neurofibroma Development 

Little has been reported about a possible role of miRNAs on neurofibroma development. After a study aimed at comprehensively characterizing the expression pattern of 377 miRNAs in NF1-related neurofibromas, Masliah-Planchon et al. demonstrated that miR-486-3p was the most significantly upregulated miRNA in PNFs. Interestingly, *PTEN*, a tumor suppressor gene, is a target of miR-486-3p. Accordingly, the authors hypothesized that in PNF development, miR-486-3p may be a major onco-miR, downregulating *PTEN*. Aberrant expression of miRNAs involved in the RAS-MAPK pathway, such as miR-370, miR-143, miR-181a, and miR-145, has been also reported. Further studies should be performed to assess the implication of these miRNAs in neurofibroma generation [21]. 

**Figure 1:**
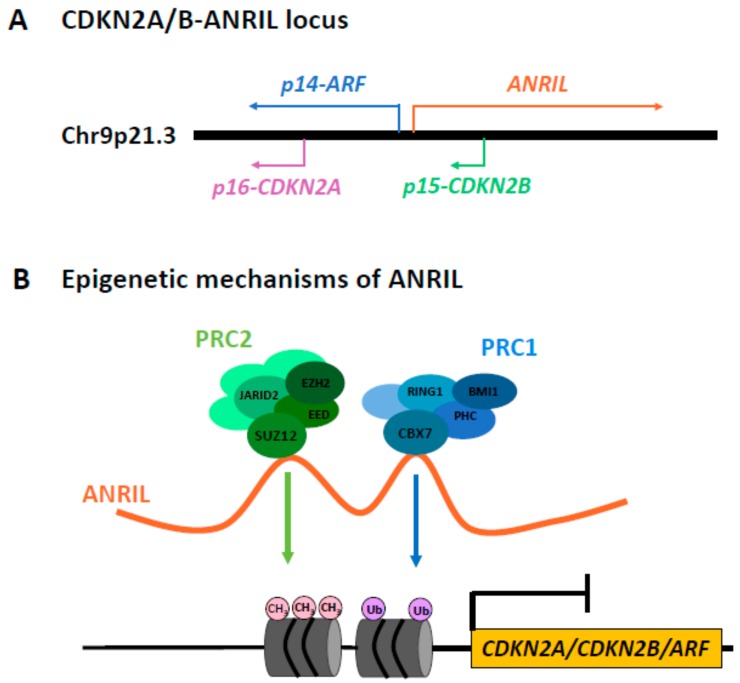
(**a**) Genomic organization of the human *CDKN2A/B* locus on chromosome 9p21.3, encoding for the three tumor suppressors p14-ARF, p15-CDKN2B, p16-CDKN2A, and for the long non-coding RNA (lncRNA) ANRIL. *ANRIL* partially overlaps *CDKN2B*, and is transcribed in the antisense orientation of the *CDKN2B–CDKN2A–ARF* gene cluster. (**b**) The direct interaction between *ANRIL* and the polycomb repressive complexes 1 and 2 (PRC1 and PRC2) allows maintenance of the transcriptional repression of the *CDKN2A/B* locus, via a *cis*-acting, polycomb-mediated epigenetic mechanism. PRC1 and PRC2 catalyse the mono-ubiquitination and the methylation of the histone code about the *CDKN2A/B* locus, respectively.

As far as the long non-coding RNAs, the only one currently related to tumorigenesis in patients affected by NF1 is ANRIL, which seems to be associated with PNF development [13]. PNFs are protruding and deforming masses that arise from multiple nerves, and also involve connective tissue and skin folds, displayed by 30% of NF1 patients [48]. *ANRIL* is an anti-sense, long-non-coding RNA expressed by *CDKN2B-AS1* in the *INK4* locus, located on chromosome 9p21 (Figure 1a). 

*ANRIL* is involved in the expression regulation of three tumor suppressor genes at the *CDKN2A/B* locus (*p16-CDKN2A*, *p15-CDKN2B*, and *p14-ARF*), via a *cis*-acting, polycomb-mediated epigenetic mechanism. This lncRNA stabilizes the polycomb repressive complexes 1 and 2 (PRC1 and PRC2), acting as a recruiting platform for their specific subunits (CBX7 of PRC1 and SUZ12 of PRC2). The direct interaction between *ANRIL* and PRC1/PRC2 allows these molecular complexes to modify the histone code of the *CDKN2A/B* locus, maintaining the transcriptional repression [49,50]. PRC1 and PRC2 catalyse the mono-ubiquitination of H2A on K119 and the methylation of histone H3 lysine 27, respectively [51] (Figure 1b). 

Alterations of *ANRIL* expression were correlated with several human tumors and diseases. For example, *ANRIL* overexpression was found in bladder cancer [52], prostate carcinoma [50], ovarian cancer [53], cervical cancer [54], breast cancer [55], gastric cancer [56], esophageal squamous cell carcinoma [57], lung cancer [58], and hepatocellular carcinoma [59]. 

As far as the molecular characterization of *ANRIL*, both SNPs and structural alterations of this gene have been reported. Multiple SNPs in the 9p21.3 locus were associated with risk for numerous diseases, including diabetes, stroke, coronary heart disease, melanoma, and glioma [60], while structural alterations of *ANRIL*, such as deletions and translocations, were identified in neurofibromas [13] and melanomas [61].

Pasmant and colleagues identified a recurrent somatic alteration in PNFs, the 9p21.3 deletion (including the *CDKN2A/B-ANRIL* locus), in 6 out of 22 PNF tumors from 18 NF1 patients, analysed by a genome-wide array of comparative genomic hybridization. A family-based association test (FBAT) by SNP analysis of 9p21.3 region was performed, in order to assess the role of this chromosome locus in the onset and number of PNFs in NF1 patients. The FBAT study revealed a statistically significant association of the rs2151280 SNP T allele, located within the *ANRIL* intron 3, with the onset of a higher number of PNFs (*p* < 0.001) in NF1 patients, according to a dominant model. Furthermore, after *CDKN2A*, *CDKN2B*, *ARF*, and *ANRIL* expression analysis in 124 NF1 patients’ peripheral blood, the authors found that the rs2151280 T allele was significantly associated with reduced *ANRIL* transcript levels (*p* < 0.001 by Kruskal–Wallis test), suggesting a functional role of this SNP in the modulation of *ANRIL* expression that could promote PNF susceptibility in NF1 patients [13]. 

Another study carried out on a cohort of 29 NF1 microdeletion patients showed that neither the PNF number nor PNF volume were correlated with the rs2151280 T allele. Differently, in these patients PNF susceptibility was associated with the loss of one allele of *SUZ12* coding for the PRC2 subunit that interacts directly with ANRIL. As *SUZ12* is included in the 1.4 Mb NF1 microdeletion region, and because all patients enrolled for the study have only one copy of *SUZ12*, these authors hypothesize that the heterozygous constitutional deletion of this gene may influence the ANRIL-mediated expression regulation of the *CDKN2A/CDKN2B* tumor suppressor genes [62]. 

### 3.3. The *ANRIL* Rs2151280 is a Susceptibility Marker for Optic Glioma Development and Mild Phenotype in NF1 Patients

Even if the implication of lnc-RNA *ANRIL* in PNF development has not yet been assessed, different findings indicate that this issue should be further investigated, a challenge that could provide useful diagnostic markers or pharmacological targets.

To unravel this matter, we studied the occurrence of specific rs2151280 genotypes in a cohort of 104 NF1 patients, enrolled at the C. Besta Neurologic Institute, Milan, Italy. Furthermore, we determined the *CDKN2A*, *CDKN2B*, *ARF*, and *ANRIL* expression levels in the PBMCs of NF1 patients with a PNF. Table 1 summarises the clinical and molecular features of our cohort, while clinical details are illustrated in Table S1. 

Because *ANRIL* is considered a susceptibility locus for several cancers, including glioma [63], we also studied a possible association of the rs2151280 genotypes or the expression level of the above genes with the development of other tumors often associated with NF1, such as optic glioma (Table S1). The obtained results have been correlated with the types of tumors developed, with Riccardi and Ablon scores [16,17] (Table 1 and Tables S1 and S2).

We analyzed the frequency of rs2151280 alleles/genotypes in our cohort by comparing them with those of the healthy population, also considering the allele or genotype distribution within NF1 patient subgroups showing DNFs, PNFs, OPGs, or other tumors (e.g., astrocytomas and MPNSTs). After the constitutional DNA analysis, a significantly different genotype distribution (0.2 > *p* > 0.1) was observed between the OPG subgroup of NF1 patients and the European population (1000GENOMES Phase3-V1 EUR population build 14; Table 2). Interestingly, if we consider the genotype frequency within our cohort of NF1 patients, those patients with a CC genotype had a threefold higher risk of OPG development versus patients with other rs2151280 genotypes (i.e., CT; TT). The CC genotype was detected in 41.7% of patients with an OPG, and in 17.5% of patients without (*p* = 0.014; OR: 3.4; 95% CI, 1.1–10.1). No difference was observed for genotypes CT and TT distribution between patients with and without OPGs (CT: 41.7 vs. 63.7, respectively; TT: 16.7 vs. 18.8, respectively). No significant effect for sex or age was found in the regression analysis. OPG patients with a CC genotype show a wide spectrum of *NF1* mutations, which was also true if we considered the whole OPG group, suggesting an absence of correlation between a specific *NF1* mutation and OPG onset in these patients.

When Riccardi and Ablon scales were considered, only patients older than 19 years were included in the analysis, due to age-dependent diseases. A significant association between genotype TT and the presence of a mild disease was detected: the TT genotype was detected in 50% patients with Ablon 1, and in 15% with a higher Ablon scale (*p* = 0.034; OR 5.67; 95% CI, 0.90–34.08).

**Figure 2:**
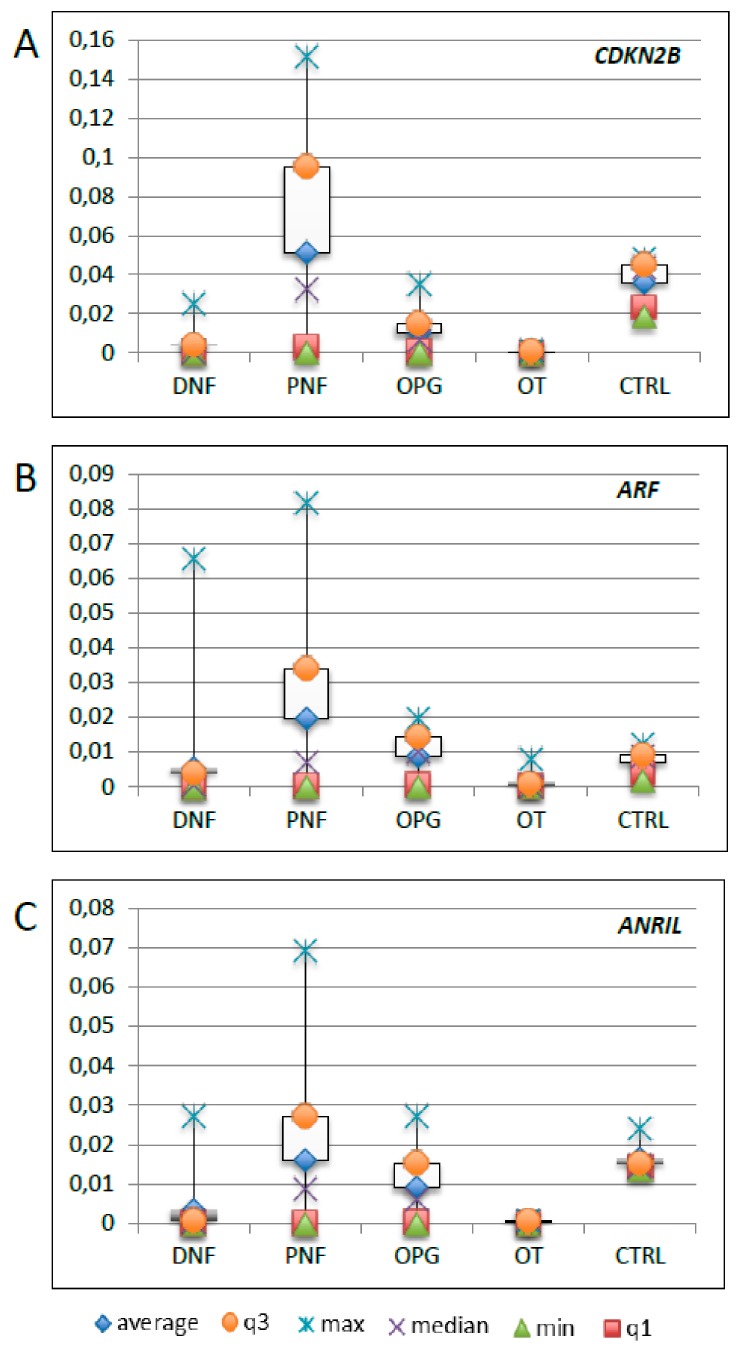
Distribution of *CDKN2B* (**a**), *ARF* (**b**), and *ANRIL* (**c**) gene expression in patients’ peripheral blood mononuclear cells (PBMCs) reported in a box plot. DNF: dermal neurofibroma; PNF: plexiform neurofibroma; OPG: optic pathway glioma; OT: other tumors; CTRL: unaffected controls.

We also investigated the expression of *ANRIL*, and its target genes *CDKN2A*, *CDKN2B*, and *ARF*, on PBMC RNAs from 65 patients of our NF1 cohort. As the expression of *CDKN2A* in the peripheral blood was very low in all samples and in the unaffected controls, it has not been reported in the results shown below. The cohort was divided into four subgroups according to the type of tumor developed by each patient, and was compared to five unrelated controls. Patients who developed multiple types of tumors were included in different sub-categories (Figure 2a–c). In general, patients with a PNF showed considerable variability, as well as those with a DNF for *ARF* (Figure 2b). The average expression levels of *CDKN2B*, *ARF*, and *ANRIL* were generally lower in NF1 patients, with the exception of PNF patients, with respect to the controls (Figure S1).

**Figure 3:**
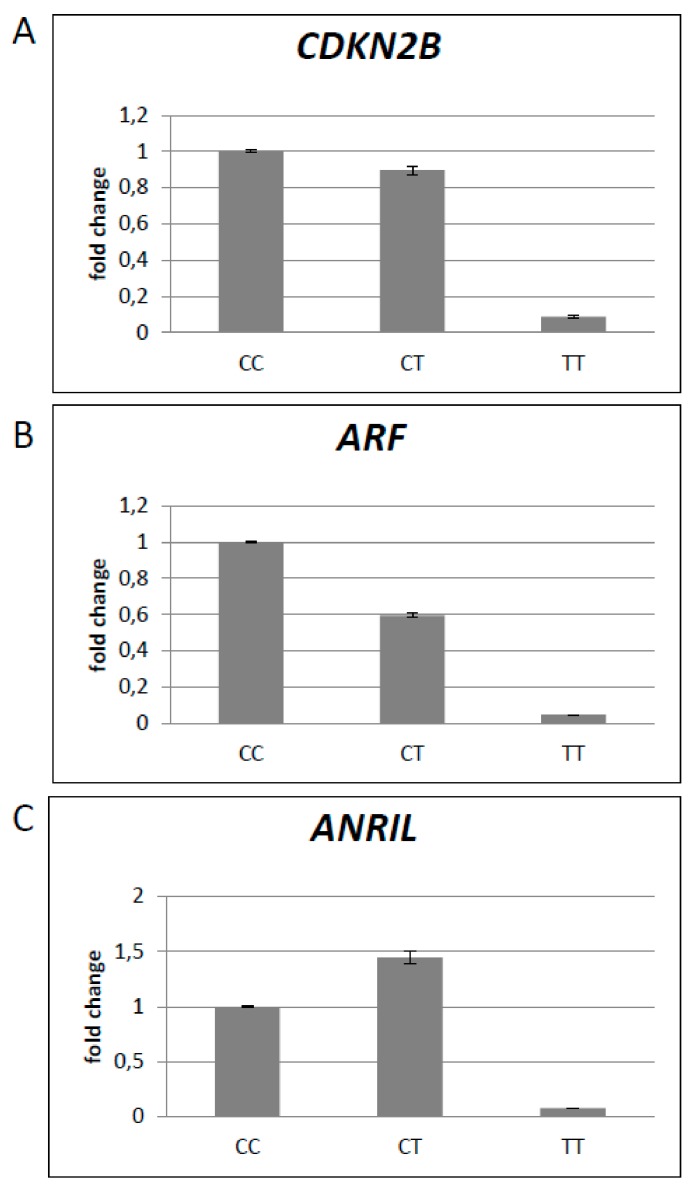
Average of the expression of *CDKN2B*, *ARF*, and *ANRIL* the PBMCs of patients who had developed an optic pathway glioma (OPG). For each gene, patients were clustered by rs2151280 genotype: DNF (**a**), PNF (**b**), OPG (**c**).

As the T-allele of rs2151280 was associated with a reduced level of *ANRIL* expression in PBMCs, each group of patients was furtherly subdivided according to the genotype of rs2151280 SNP (i.e., homozygous CC, TT, or heterozygous CT). We found that patients with an OPG carrying the TT genotype showed reduced *ANRIL* expression compared to the other subgroup of genotypes (CT and CC) (Figure 3), confirming the association previously reported [12]. Interestingly, most OPG patients carrying a CC or CT genotype did not show decreased *ANRIL* expression in the blood. These results are consistent with the biological effect of *ANRIL* expression in glioma cell lines [14]. *ANRIL* was found to be upregulated in glioma cell lines, and its silencing led to the inhibition of cell proliferation, migration, and invasion. *ANRIL* downregulation has an effect on the upregulation of miR-34a that, targeting Sirt1, mediates that downregulation, leading to the inactivation of the PI3K/AKT and mTOR pathways [14].

Furthermore, the silencing or homozygous deletion of *CDKN2A*, *ARF*, and *CDKN2B* has been detected in a subset of PNF, atypical neurofibromas, as well as MPNSTs, indicating that these genes have a role not only in the formation of PNFs, but probably also in MPNST development [13,64]. Nevertheless, this association was not observed in patients with NF1 microdeletion [62]. These data are consistent with our results on *NF1* and the *INK4b*/*ARF*/*INK4a* locus LOH studies (Figure S2). The occurrence of LOH of both the *NF1* and *INK4b*/*ARF*/*INK4a* regions has been investigated in tumor specimens of a small subset of our NF1 patients, and was reported for the first time in DNF, PNF, and astrocytoma, indicating that the genes included in the deletion interval may have a role in the tumorigenesis progression. Accordingly, the *INK4b*/*ARF*/*INK4a* locus is deleted or downregulated in about 40% of human cancers, mainly related to the tumor suppressive function of CDKN2A and CDKN2B [65]; conversely, *ANRIL* itself shows a pro-oncogenic activity [63].

Finally, the association between the rs2151280 TT genotype and mild NF1 phenotypes supports the hypothesis of a prognostic significance of this *ANRIL* polymorphism being associated with a decreased *ANRIL* expression in blood [66].

## 4. Conclusions

This review points out how research concerning the role of ncRNAs in NF1 pathogenesis is still only beginning, and aims to promote interest in this field, starting from the current knowledge, in order to open new perspectives on diagnosis and therapies. In the last decade, RNA-based technologies have allowed new approaches to set up early diagnosis, follow-up, and therapeutic strategies, which would positively impact the quality of cures and life of affected patients in the near future. From this view, is crucial to provide new insights unravelling the complex networks of ncRNAs to develop advanced technologies for treatment and diagnosis of different diseases. 

In the era of precision medicine, when dealing with a cancer-prone syndrome like NF1, new technologies, such as ncRNA-based liquid biopsies, cannot be ignored, and should be developed for screening and early detection of malignant complications. NcRNA-based research may underpin new therapeutic approaches and help in unraveling the issue of the low genotype/phenotype correlation in NF1.

From this perspective, our findings suggest that *ANRIL* may be a potential prognostic marker in the NF1 disease. The rs2151280 CC genotype is associated with an increased risk of OPG onset in NF1 patients, and the TT genotype is a predictive marker of a mild clinical phenotype. Although OPG generally has an indolent course, compared to non-NF1 cases, and the survival rate is good, it can sometimes cause severe visual impairment. Considering that the usefulness of brain MRI screening for the early diagnosis of optic glioma in children with NF1 is still controversial, the identification of a predictive marker may provide personalized management and follow-up for NF1 patients, with definite positive repercussions for people’s quality of life and for communities’ health social costs. 

## Figures and Tables

**Table 1:** Clinical and molecular features of the analyzed.

Clinical Characterization	N° (%)
N° pts	104
Gender (M/F)	42/62
Median Age (Range)	35 (2–71)
DNF	91/104 (86)
PNF	39/104 (38)
OPG	24/104 (23)
Other CNS Tumors	17/104 (16)
Extra CNSTumors	15/104 (14)
**Clinical scores**	**N° (%)**
Riccardi Score 1	9/104 (9)
Riccardi Score 2	32/104(31)
Riccardi Score 3	24/104 (23)
Riccardi Score 4	39/104 (38)
Ablon Score 1	17/104 (16)
Ablon Score 2	39/104 (38)
Ablon Score 3	33/104 (32)
Ablon Score 4	15/104 (14)
**Molecular Characterization**	**N° (%)**
Mutation detected	100/104 (96)
Point mutation	58/104 (56)
Intragenic deletion/duplication	42/104 (40)

DNF: dermal neurofibroma; PNF: plexiform neurofibroma; OPG: optic pathway glioma; CNS: central nervous system.

**Table 2:** Rs2151280 genotype frequency distribution in patients’ subgroups, according to the tumor type.

SAMPLES	f(C)	f(T)	f(CC)	f(CT)	f(TT)
Complete casuistry (*n* = 104)	0.52	0.48	(n = 24) 0.23	(n = 61) 0.59	(n = 19) 0.18
DNF (*n* = 91)	0.51	0.49	(n = 19) 0.21	(n = 56) 0.62	(n = 16) 0.18
PNF (*n* = 39)	0.54	0.46	(n = 9) 0.23	(n = 24) 0.62	(n = 6) 0.15
OPG (*n* = 24)	0.62	0.38	(n = 10) 0.42	(n = 10) 0.42	(n = 4) 0.17
Other tumors (*n* = 17)	0.56	0.44	(n = 4) 0.24	(n = 10) 0.59	(n = 3) 0.18
Unaffected Controls	0.5	0.5	0.2	0.6	0.2
General population	0.5	0.5	0.27	0.46	0.27

DNF: dermal neurofibroma; PNF: plexiform neurofibroma; OPG: optic pathway glioma.

## Supplementary Materials

The following are available online at https://www.mdpi.com/2073-4425/10/11/892/s1, Figure S1: Average of the expression of CDKN2B, ARF, and *ANRIL* in patients’ PBMCs. Patients were clustered for the type of tumor they had developed: DNF (**a**), PNF (**b**), OPG (**c**), or OT (**d**). * *p* < 0.5; ** *p* < 0.01; *** *p* <0.001, Figure S2: LOH analysis in *NF1* locus (**a**) and *CDKN2A/ARF* locus (**b**). Black square indicates presence of LOH; empty square indicate absence of LOH—uninformative marker; n.d.: undetermined; CEN: centromere; TEL: telomere, Table S1: Clinical and genotyping data of the analyzed NF1 cohort, Table S2: Measures of disease severity, Table S3: Primers’ sequences, Table S4: MiRNA expression level in tumors from NF1 patients.

## References

[1] Anastasiadou E., Jacob L.S., Slack F.J. (2017). Non-coding RNA networks in cancer. Nat. Rev. Cancer.

[2] Bejerano G., Pheasant M., Makunin I., Stephen S., Kent W.J., Mattick J.S., Haussler D. (2004). Ultraconserved elements in the human genome. Science.

[3] Johnsson P., Lipovich L., Grandér D., Morris K.V. (2014). Evolutionary conservation of long non-coding RNAs; Sequence, structure, function. Biochim. Biophys. Acta-Gen. Subj..

[4] Djebali S., Davis C.A., Merkel A., Dobin A., Lassmann T., Mortazavi A., Tanzer A., Lagarde J., Lin W., Schlesinger F. (2012). Landscape of transcription in human cells. Nature.

[5] Feingold E.A., Good P.J., Guyer M.S., Kamholz S., Liefer L., Wetterstrand K., Collins F.S., Gingeras T.R., Kampa D., Sekinger E.A. (2004). The ENCODE (ENCyclopedia of DNA Elements) Project. Science.

[6] Ebert M.S., Sharp P.A. (2012). Roles for microRNAs in conferring robustness to biological processes. Cell.

[7] Yamamura S., Imai-Sumida M., Tanaka Y., Dahiya R. (2018). Interaction and cross-talk between non-coding RNAs. Cell. Mol. Life Sci..

[8] Moncini S., Bevilacqua A., Venturin M., Fallini C., Ratti A., Nicolin A., Riva P. (2007). The 3′ untranslated region of human Cyclin-Dependent Kinase 5 Regulatory subunit 1 contains regulatory elements affecting transcript stability. BMC Mol. Biol..

[9] Zuccotti P., Colombrita C., Moncini S., Barbieri A., Lunghi M., Gelfi C., De Palma S., Nicolin A., Ratti A., Venturin M. (2014). HnRNPA2/B1 and nELAV proteins bind to a specific U-rich element in CDK5R1 3’-UTR and oppositely regulate its expression. Biochim. Biophys. Acta-Gene Regul. Mech..

[10] Upadhyaya M. (2011). Genetic basis of tumorigenesis in NF1 malignant peripheral nerve sheath tumors. Front. Biosci..

[11] Sedani A., Cooper D.N., Upadhyaya M. (2012). An emerging role for microRNAs in NF1 tumorigenesis. Hum. Genom..

[12] Pasmant E., Sabbagh A., Vidaud M., Bièche I. (2011). ANRIL, a long, noncoding RNA, is an unexpected major hotspot in GWAS. FASEB J..

[13] Pasmant E., Sabbagh A., Masliah-Planchon J., Ortonne N., Laurendeau I., Melin L., Ferkal S., Hernandez L., Leroy K., Valeyrie-Allanore L. (2011). Role of noncoding RNA ANRIL in genesis of plexiform neurofibromas in neurofibromatosis type 1. J. Natl. Cancer Inst..

[14] Dong X., Jin Z., Chen Y., Xu H., Ma C., Hong X., Li Y., Zhao G. (2018). Knockdown of long non-coding RNA ANRIL inhibits proliferation, migration, and invasion but promotes apoptosis of human glioma cells by upregulation of miR-34a. J. Cell. Biochem..

[15] Griffiths S., Thompson P., Frayling I., Upadhyaya M. (2007). Molecular diagnosis of neurofibromatosis type 1, 2 Years experience. Fam. Cancer.

[16] Ablon J. (1996). Gender response to neurofibromatosis. Soc. Sci. Med..

[17] Riccardi V.M., Riccardi S.L. (1982). Von Recklinghausen neurofibromatosis: New perspectives. Tex. Med..

[18] Ferrari L., Scuvera G., Tucci A., Bianchessi D., Rusconi F., Menni F., Battaglioli E., Milani D., Riva P. (2017). Identification of an atypical microdeletion generating the *RNF135-SUZ12* chimeric gene and causing a position effect in an NF1 patient with overgrowth. Hum. Genet..

[19] Lee W., Teckie S., Wiesner T., Ran L., Prieto Granada C.N., Lin M., Zhu S., Cao Z., Liang Y., Sboner A. (2014). PRC2 is recurrently inactivated through *EED* or *SUZ12* loss in malignant peripheral nerve sheath tumors. Nat. Genet..

[20] Staedtke V., Bai R.Y., Blakeley J.O. (2017). Cancer of the peripheral nerve in neurofibromatosis type 1. Neurotherapeutics.

[21] Masliah-Planchon J., Pasmant E., Luscan A., Laurendeau I., Ortonne N., Hivelin M., Varin J., Valeyrie-Allanore L., Dumaine V., Lantieri L. (2013). MicroRNAome profiling in benign and malignant neurofibromatosis type 1-associated nerve sheath tumors: Evidences of PTEN pathway alterations in early NF1 tumorigenesis. BMC Genom..

[22] Johnson S.M., Grosshans H., Shingara J., Byrom M., Jarvis R., Cheng A., Labourier E., Reinert K.L., Brown D., Slack F.J. (2005). RAS is regulated by the let-7 microRNA family. Cell.

[23] Chai G., Liu N., Ma J., Li H., Oblinger J.L., Prahalad A.K., Gong M., Chang L.S., Wallace M., Muir D. (2010). MicroRNA-10b regulates tumorigenesis in neurofibromatosis type 1. Cancer Sci..

[24] Li D., Zhao Y., Liu C., Chen X., Qi Y., Jiang Y., Zou C., Zhang X., Liu S., Wang X. (2011). Analysis of MiR-195 and MiR-497 expression, regulation and role in breast cancer. Clin. Cancer Res..

[25] Lewis B.P., Shih I., Jones-Rhoades M.W., Bartel D.P., Burge C.B. (2003). Prediction of mammalian microRNA targets. Cell.

[26] Poliseno L., Salmena L., Riccardi L., Fornari A., Song M.S., Hobbs R.M., Sportoletti P., Varmeh S., Egia A., Fedele G. (2010). Identification of the *miR-106b*∼*25* microRNA cluster as a proto-oncogenic *PTEN*-targeting intron that cooperates with its host gene *MCM7* in transformation. Sci. Signal..

[27] Shi W., Gerster K., Alajez N.M., Tsang J., Waldron L., Pintilie M., Hui A.B., Sykes J., P’ng C., Miller N. (2011). MicroRNA-301 mediates proliferation and invasion in human breast cancer. Cancer Res..

[28] Yong S.L., Dutta A. (2007). The tumor suppressor microRNA *let-7* represses the HMGA2 oncogene. Genes Dev..

[29] Ma L., Young J., Prabhala H., Pan E., Mestdagh P., Muth D., Teruya-Feldstein J., Reinhardt F., Onder T.T., Valastyan S. (2010). MiR-9, a MYC/MYCN-activated microRNA, regulates E-cadherin and cancer metastasis. Nat. Cell Biol..

[30] Nagel R., Le Sage C., Diosdado B., Van Der Waal M., Oude Vrielink J.A.F., Bolijn A., Meijer G.A., Agami R. (2008). Regulation of the adenomatous polyposis coli gene by the miR-135 family in colorectal cancer. Cancer Res..

[31] Peter M.E. (2009). Let-7 and miR-200 microRNAs: Guardians against pluripotency and cancer progression. Cell Cycle.

[32] Rotkrua P., Akiyama Y., Hashimoto Y., Otsubo T., Yuasa Y. (2011). MiR-9 downregulates CDX2 expression in gastric cancer cells. Int. J. Cancer.

[33] Han L., Witmer P.D.W., Casey E., Valle D., Sukumar S. (2007). DNA methylation regulates microRNA expression. Cancer Biol. Ther..

[34] Yekta S., Shih I.H., Bartel D.P. (2004). MicroRNA-directed cleavage of HOXB8 mRNA. Science.

[35] Huang X., Ding L., Bennewith K.L., Tong R.T., Welford S.M., Ang K.K., Story M., Le Q.T., Giaccia A.J. (2009). Hypoxia-inducible mir-210 regulates normoxic gene expression involved in tumor initiation. Mol. Cell.

[36] Schultz J., Lorenz P., Gross G., Ibrahim S., Kunz M. (2008). MicroRNA let-7b targets important cell cycle molecules in malignant melanoma cells and interferes with anchorage-independent growth. Cell Res..

[37] Trompeter H.I., Abbad H., Iwaniuk K.M., Hafner M., Renwick N., Tuschl T., Schira J., Müller H.W., Wernet P. (2011). MicroRNAs MiR-17, MiR-20a, and MiR-106b Act in concert to modulate E2F activity on cell cycle arrest during neuronal lineage differentiation of USSC. PLoS ONE.

[38] Wu J., Qian J., Li C., Kwok L., Cheng F., Liu P., Perdomo C., Kotton D., Vaziri C., Anderlind C. (2010). miR-129 regulates cell proliferation by downregulating Cdk6 expression. Cell Cycle.

[39] Xu T., Zhu Y., Xiong Y., Ge Y.Y., Yun J.P., Zhuang S.M. (2009). MicroRNA-195 suppresses tumorigenicity and regulates G 1 /S transition of human hepatocellular carcinoma cells. Hepatology.

[40] Subramanian S., Thayanithy V., West R.B., Lee C.H., Beck A.H., Zhu S., Downs-Kelly E., Montgomery K., Goldblum J.R., Hogendoorn P.C.W. (2010). Genome-wide transcriptome analyses reveal p53 inactivation mediated loss of miR-34a expression in malignant peripheral nerve sheath tumours. J. Pathol..

[41] Lee Y.B., Bantounas I., Lee D.Y., Phylactou L., Caldwell M.A., Uney J.B. (2009). Twist-1 regulates the miR-199a/214 cluster during development. Nucleic Acids Res..

[42] Gong M., Ma J., Li M., Zhou M., Hock J.M., Yu X. (2012). MicroRNA-204 critically regulates carcinogenesis in malignant peripheral nerve sheath tumors. Neuro Oncol..

[43] Itani S., Kunisada T., Morimoto Y., Yoshida A., Sasaki T., Ito S., Ouchida M., Sugihara S., Shimizu K., Ozaki T. (2012). MicroRNA-21 correlates with tumorigenesis in malignant peripheral nerve sheath tumor (MPNST) via programmed cell death protein 4 (PDCD4). J. Cancer Res. Clin. Oncol..

[44] Presneau N., Eskandarpour M., Shemais T., Henderson S., Halai D., Tirabosco R., Flanagan A.M. (2013). MicroRNA profiling of peripheral nerve sheath tumours identifies miR-29c as a tumour suppressor gene involved in tumour progression. Br. J. Cancer.

[45] Listernick R., Louis D.N., Packer R.J., Gutmann D.H. (1997). Optic pathway gliomas in children with neurofibromatosis 1, Consensus statement from the NF1 optic pathway glioma task force. Ann. Neurol..

[46] Ho C.Y., Bar E., Giannini C., Marchionni L., Karajannis M.A., Zagzag D., Gutmann D.H., Eberhart C.G., Rodriguez F.J. (2013). MicroRNA profiling in pediatric pilocytic astrocytoma reveals biologically relevant targets, including PBX3, NFIB, and METAP2. Neuro Oncol..

[47] Darrigo Júnior L.G., Lira R.C.P., Fedatto P.F., Marco Antonio D.S., Valera E.T., Aguiar S., Yunes J.A., Brandalise S.R., Neder L., Saggioro F.P. (2019). MicroRNA profile of pediatric pilocytic astrocytomas identifies two tumor-specific signatures when compared to non-neoplastic white matter. J. Neurooncol..

[48] Tchernev G., Chokoeva A.A., Patterson J.W., Bakardzhiev I., Wollina U., Tana C. (2016). Plexiform Neurofibroma: A Case Report. Medicine (Baltimore).

[49] Drak Alsibai K., Vacher S., Meseure D., Nicolas A., Lae M., Schnitzler A., Chemlali W., Cros J., Longchampt E., Cacheux W. (2019). High positive correlations between ANRIL and p16-CDKN2A/p15-CDKN2B/p14-ARF gene cluster overexpression in multi-tumor types suggest deregulated activation of an ANRIL–ARF bidirectional promoter. Non-Coding RNA.

[50] Yap K.L., Li S., Muñoz-Cabello A.M., Raguz S., Zeng L., Mujtaba S., Gil J., Walsh M.J., Zhou M.M. (2010). Molecular interplay of the noncoding RNA *ANRIL* and methylated histone H3 lysine 27 by polycomb CBX7 in transcriptional silencing of *INK4a*. Mol. Cell.

[51] Margueron R., Li G., Sarma K., Blais A., Zavadil J., Woodcock C.L., Dynlacht B.D., Reinberg D. (2008). Ezh1 and Ezh2 maintain repressive chromatin through different mechanisms. Mol. Cell.

[52] Zhu H., Li X., Song Y., Zhang P., Xiao Y., Xing Y. (2015). Long non-coding RNA ANRIL is up-regulated in bladder cancer and regulates bladder cancer cell proliferation and apoptosis through the intrinsic pathway. Biochem. Biophys. Res. Commun..

[53] Qiu J.J., Lin Y.Y., Ding J.X., Feng W.W., Jin H.Y., Hua K.Q. (2015). Long non-coding RNA ANRIL predicts poor prognosis and promotes invasion/metastasis in serous ovarian cancer. Int. J. Oncol..

[54] Naemura M., Murasaki C., Inoue Y., Okamoto H., Kotake Y. (2015). Long noncoding RNA ANRIL regulates proliferation of non-small cell lung cancer and cervical cancer cells. Anticancer Res..

[55] Meseure D., Vacher S., Alsibai K.D., Nicolas A., Chemlali W., Caly M., Lidereau R., Pasmant E., Callens C., Bieche I. (2016). Expression of *ANRIL*-polycomb complexes-*CDKN2A/B/ARF* genes in breast tumors: Identification of a two-gene (*EZH2/CBX7*) signature with independent prognostic value. Mol. Cancer Res..

[56] Zhang E.B., Kong R., Yin D.D., You L.H., Sun M., Han L., Xu T.P., Xia R., Yang J.S., De W. (2014). Long noncoding RNA ANRIL indicates a poor prognosis of gastric cancer and promotes tumor growth by epigenetically silencing of miR-99a/miR-449a. Oncotarget.

[57] Chen D., Zhang Z., Mao C., Zhou Y., Yu L., Yin Y., Wu S., Mou X., Zhu Y. (2014). ANRIL inhibits p15INK4b through the TGFβ1 signaling pathway in human esophageal squamous cell carcinoma. Cell. Immunol..

[58] Nie F.Q., Sun M., Yang J.S., Xie M., Xu T.P., Xia R., Liu Y.W., Liu X.H., Zhang E.B., Lu K.H. (2015). Long noncoding RNA ANRIL promotes non-small cell lung cancer cell proliferation and inhibits apoptosis by silencing KLF2 and P21 expression. Mol. Cancer Ther..

[59] Huang M.D., Chen W.M., Qi F.Z., Xia R., Sun M., Xu T.P., Yin L., Zhang E.B., De W., Shu Y.Q. (2015). Long non-coding RNA ANRIL is upregulated in hepatocellular carcinoma and regulates cell apoptosis by epigenetic silencing of KLF2. J. Hematol. Oncol..

[60] Cunnington M.S., Koref M.S., Mayosi B.M., Burn J., Keavney B. (2010). Chromosome 9p21 SNPs associated with multiple disease phenotypes correlate with ANRIL expression. PLoS Genet..

[61] Pasmant E., Laurendeau I., Héron D., Vidaud M., Vidaud D., Bièche I. (2007). Characterization of a germ-line deletion, including the entire *INK4/ARF* locus, in a melanoma-neural system tumor family: Identification of *ANRIL*, an antisense noncoding RNA whose expression coclusters with *ARF*. Cancer Res..

[62] Mußotter T., Kluwe L., Högel J., Nguyen R., Cooper D.N., Mautner V.F., Kehrer-Sawatzki H. (2012). Non-coding RNA *ANRIL* and the number of plexiform neurofibromas in patients with *NF1* microdeletions. BMC Med. Genet..

[63] Kong Y., Hsieh C.-H., Alonso L.C. (2018). ANRIL: A lncRNA at the CDKN2A/B locus with roles in cancer and metabolic disease. Front. Endocrinol. (Lausanne).

[64] Beert E., Brems H., Daniëls B., de Wever I., van Calenbergh F., Schoenaers J., Debiec-Rychter M., Gevaert O., de Raedt T., van den Bruel A. (2011). Atypical neurofibromas in neurofibromatosis type 1 are premalignant tumors. Genes Chromosom. Cancer.

[65] Tano K., Akimitsu N. (2012). Long non-coding RNAs in cancer progression. Front. Genet..

[66] Kotake Y., Nakagawa T., Kitagawa K., Suzuki S., Liu N., Kitagawa M., Xiong Y. (2011). Long non-coding RNA ANRIL is required for the PRC2 recruitment to and silencing of p15 INK4B tumor suppressor gene. Oncogene.

