# Supplementary material for: Non-Coding RNA and Tumor Development in Neurofibromatosis Type 1: ANRIL Rs2151280 Is Associated with Optic Glioma Development and a Mild Phenotype in Neurofibromatosis Type 1 Patients
Source: Genes (Basel). 2019 Nov 5;10(11):892. doi: 10.3390/genes10110892 (PMC6895873; doi:10.3390/genes10110892)
Supplement: Supplementary file 1 [file genes-10-00892-s001.zip › SupplementaryRev/Figure S2.pdf]

A

CEN **17q11.2** *TEL*

NF1

patient	tumor	D17S841	D17S1294	D17S1307	D17S2163	EVI20	D17S1166	IVS27 AC33.1	IVS27 TG24.8	IVS38	D17S1800	D17S798
6 (1)	DNF	-	■	□	□	n.d.	□	-	n.d.	□	□	□
7	DNF	■	n.d.	-	-	-	-	-	■	-	■	-
10	DNF	■	n.d.	-	-	n.d.	□	■	n.d.	n.d.	■	-
11	DNF	□	n.d.	□	-	n.d.	□	□	n.d.	n.d.	□	-
13	DNF	-	■	□	-	-	-	-	-	-	■	-
3	PNF	-	■	■	-	n.d.	■	■	n.d.	n.d.	■	-
5	PNF	-	■	-	□	n.d.	■	■	n.d.	-	-	-
6 (2)	PNF	-	■	■	□	n.d.	■	-	n.d.	■	■	■
2	MPNST	-	-	-	-	n.d.	■	-	n.d.	■	-	-
8	MPNST	□	n.d.	□	-	n.d.	■	■	n.d.	n.d.	-	-
18	ATYPIC ASTROCITOMA	-	□	-	■	n.d.	□	■	n.d.	n.d.	-	-
12	PILOCYTIC ASTROCITOMA	-	■	□	-	n.d.	□	■	n.d.	n.d.	□	-
16	PILOCYTIC ASTROCITOMA	-	■	□	-	□	■	-	n.d.	-	□	-

B

TEL **9p21.3** **9p21.2** **9p21.1** *CEN*

CDKN2A/ARF

patient	tumor	D9S736	D9S974	D9S942	D9S171	D9S169	D9S104
6 (1)	DNF	■	–	■	□	■	–
7	DNF	–	–	□	□	■	–
10	DNF	–	■	■	■	□	–
11	DNF	–	■	■	–	■	□
13	DNF	–	■	■	■	–	■
3	PNF	–	■	■	□	■	■
5	PNF	–	–	■	■	□	–
6 (2)	PNF	□	–	■	□	□	–
2	MPNST	–	–	■	–	–	–
8	MPNST	–	■	■	–	■	–
18	ATYPIC ASTROCITOMA	–	■	■	–	■	–
12	PILOCYTIC ASTROCITOMA	–	□	□	□	■	–
16	PILOCYTIC ASTROCITOMA	–	–	–	■	–	–
