# Supplementary material for: Non-Coding RNA and Tumor Development in Neurofibromatosis Type 1: ANRIL Rs2151280 Is Associated with Optic Glioma Development and a Mild Phenotype in Neurofibromatosis Type 1 Patients
Source: Genes (Basel). 2019 Nov 5;10(11):892. doi: 10.3390/genes10110892 (PMC6895873; doi:10.3390/genes10110892)
Supplement: Supplementary file 1 [file genes-10-00892-s001.zip › SupplementaryRev/TableS4.docx]

**Table S4. miRNA expression level in tumors from NF1 patients**

| Tumor | miRNA | Expression level in tumor | Expression level in cell line | References |
| --- | --- | --- | --- | --- |
| MPNST  Pilocytic astrocytoma  PNF | miR-139-5p  miR-150  miR-338-3p  miR-195  miR-146a  miR-95  let-7b  miR-186  miR-885-5p  miR-200c  miR-135b  miR-449a  miR-210  miR-301b  miR-301a  miR-9  miR-130b  miR-454  miR-19a  miR-106b  miR-135a  miR-137  miR-31  miR-129-3p  miR-224  miR-10b  miR-148a  miR-18a  miR-452  miR-598  miR-196b  miR-425  miR-10a  miR-93  miR-20a  miR-19b  miR-484  miR-192  miR-34a  miR-214  miR-204  mir-21  miR-29c  miR-29c*  miR-30e*  miR-30c  miR-340*  miR-139-5p  miR-195  miR-151-5p  miR- 342-5p  miR-146a  miR-150  miR-223  let-7 a  let-7 g  miR-210  miR-339-5p  hsa-miR-744*  hsa-miR-187*  hsa-miR-650  hsa-miR-1276  hsa-miR-33b-3p  hsa-miR-149-5p  hsa-miR-923_v12.0  hsa-miR-144-3p  hsa-miR-769-5p  hsa-miR-542-5p  hsa-miR-455-5p  hsa-miR-218-5p  hsa-miR-142-5p  hsa-miR-744-5p  hsa-miR-483-5p  hsa-miR-376b  hsa-miR-199b-5p  hsa-miR-219-2-3p  hsa-miR-382-5p  hsa-miR-139-3p  hsa-miR-337-5p  hsa-miR-138-5p  hsa-miR-181c-3p  hsa-miR-136-3p  hsa-miR-125a-3p  hsa-miR-154-5p  hsa-miR-575  hsa-miR-21-3p  hsa-miR-1275  hsa-miR-135a-3p  hsa-miR-409-3p  hsa-miR-379-5p  hsa-miR-630  hsa-miR-134  hsa-miR-188-5p  miR-486-3p  miR-370  miR-143  miR-145  miR-181a | down  down  down  down  down  down  down  down  down  down  up  up  up  up  up  up  up  up  up  up  up  up  up  up  up  up  up  up  up  up  up  up  up  up  up  up  up  up  down  up  down  up  down  down  down  down  down  down  down  down  down  down  down  down  down  down  up  up  down  down  up  up  down  down  down  down  down  down  down  down  down  down  down  down  down  down  down  down  down  down  down  down  down  down  down  down  down  down  down  down  down  down  up  up  up  up  up  up | down  down  up  down | [21]  [21]  [21]  [21]  [21]  [21]  [21]  [21]  [21]  [21]  [21]  [21]  [21]  [21]  [21]  [21]  [21]  [21]  [21]  [21]  [21]  [21]  [21]  [21]  [21]  [21]  [21]  [21]  [21]  [21]  [21]  [21]  [21]  [21]  [21]  [21]  [21]  [21]  [40]  [40]  [42]  [43]  [44]  [44]  [44]  [44]  [44]  [44]  [44]  [44]  [44]  [44]  [44]  [44]  [44]  [44]  [44]  [44]  [46]  [46]  [46]  [46]  [47]  [47]  [47]  [47]  [47]  [47]  [47]  [47]  [47]  [47]  [47]  [47]  [47]  [47]  [47]  [47]  [47]  [47]  [47]  [47]  [47]  [47]  [47]  [47]  [47]  [47]  [47]  [47]  [47]  [47]  [47]  [21]  [21]  [21]  [21]  [21] |
