# Supplementary figures and images for: Non-Coding RNA and Tumor Development in Neurofibromatosis Type 1: ANRIL Rs2151280 Is Associated with Optic Glioma Development and a Mild Phenotype in Neurofibromatosis Type 1 Patients
Source: Genes (Basel). 2019 Nov 5;10(11):892. doi: 10.3390/genes10110892 (PMC6895873; doi:10.3390/genes10110892)

A

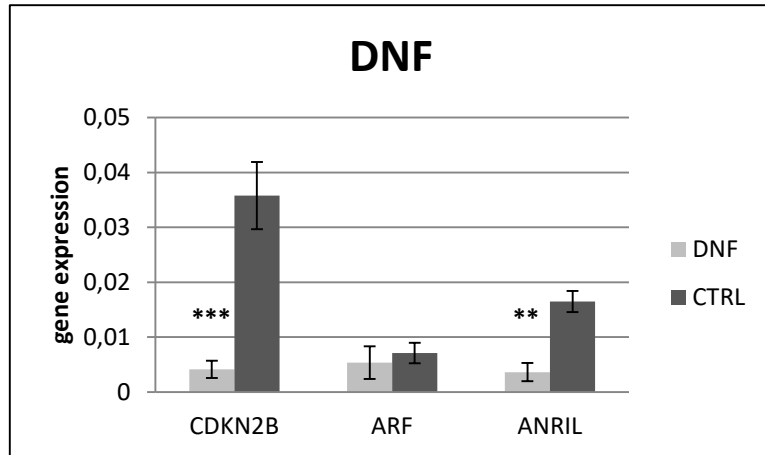

C

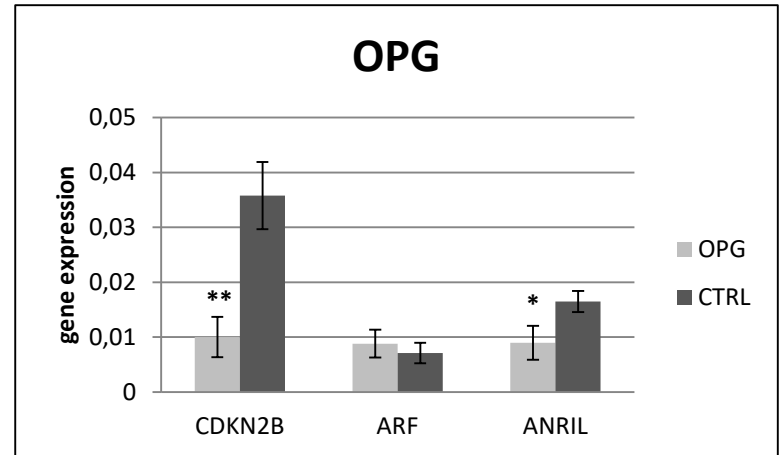

B

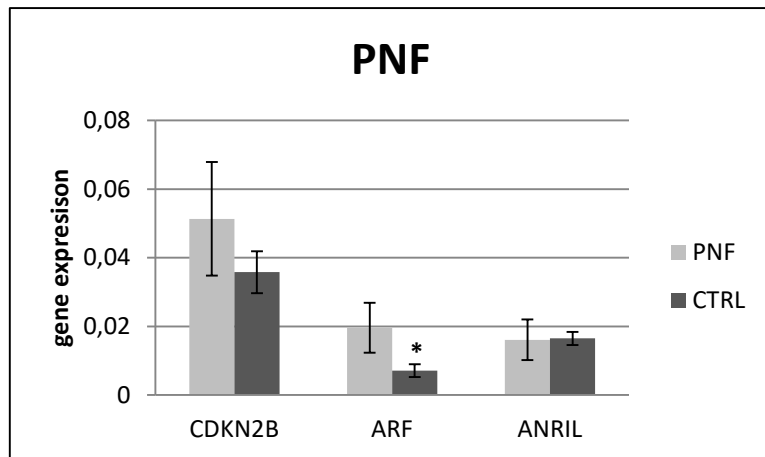

D

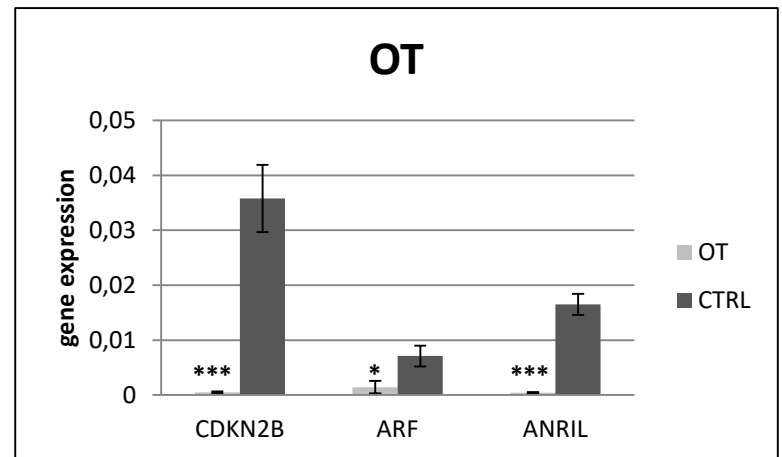

Supplement: Supplementary file 1 [file genes-10-00892-s001.zip › SupplementaryRev/Figure S1.pdf]
